# The Effect of Different Condition of Pulpal Pressure on Microtensile Bond Strength of Several Dentin Bonding Agents on Deep and Superficial Dentin

**DOI:** 10.3390/ma14206200

**Published:** 2021-10-19

**Authors:** Marco Montanari, Luca Fiorillo, Gabriele Cervino, Sergio Sambataro, Alan Scott Herford, Marco Cicciù

**Affiliations:** 1 Department of Prosthodontics, University of Ferrara, Via Luigi Borsari 46, 44121 Ferrara, Italy; docmontanari.marco@gmail.com; 2 Department of Biomedical and Dental Sciences, Morphological and Functional Images, University of Messina, Policlinico G. Martino, Via Consolare Valeria, 98100 Messina, Italy; gcervino@unime.it (G.C.); ssambataro@centrodiortodonzia.it (S.S.); mcicciu@unime.it (M.C.); 3 Multidisciplinary Department of Medical-Surgical and Dental Specialties, Second University of Naples, 80100 Naples, Italy; 4 Department of Dentistry, University of Aldent, 1000 Tirana, Albania; 5 Department of Maxillofacial Surgery, Loma Linda University, Loma Linda, CA 92354, USA; aherford@llu.edu

**Keywords:** pulpal pressure, microtensile bond strength, permeability, hydrophilic monomers, hybrid layer, dentin bonding systems, SEM

## Abstract

The objective of this study was to examine the effect of different conditions of simulated hydrostatic pulpal pressure on the μTBS of HEMA-based and HEMA-free dentin bonding agents (DBAs). The influence of dentin location (deep and superficial) on μTBS was also evaluated. Flat coronal dentin surfaces of extracted human molars were prepared. Three groups of resin-bonded specimens were exposed to different pulpal pressures. Pulpal pressure was maintained for 20 min for each group. A flowable resin composite was used for coronal build-up. The bonded teeth were sectioned and, after 24 h of water storage, stressed to failure using the microtensile tester (μTBS). Failed samples were analyzed by SEM inspection. HEMA-based DBAs were much more sensitive to pulpal pressure conditions than non-HEMA-containing DBAs. Pulpal pressure had a greater influence in deep dentin. The HEMA-free DBA was insensitive to the presence or absence of pulpal pressure condition. SEM inspection confirmed a relationship between the presence of voids inside the HEMA-based DBAs layer and the lower μTBS results. HEMA-based DBAs are more sensitive to pulpal pressure conditions than HEMA-free DBAs. Interestingly, HEMA-free DBA showed a greater number of water droplets at resin–dentin interface in all tested conditions.

## 1. Introduction

Intrapulpal pressure and regional variations in dentinal tubule density are main factors that regulate the volume of intrinsic water present during the dentin bond establishment. Bonding to deep dentin has been more challenging than bonding to superficial dentin [1,2], mainly due to the reduced area of solid intertubular dentin [3] associated with the increase water content. Likewise, positive intrapulpal pressure has been regarded to be detrimental to the bonding process [4,5,6]. Lower bond strengths are produced in the presence of positive intrapulpal pressure because of the increase in water outflow to the surface of dentin [7,8]. Transudation of fluid droplets across polymerized adhesives bonded to dentin has been observed both in vitro and in vivo [9,10,11]. The outward movement of dentinal fluid under a slight positive pulpal pressure seems to permeate polymerized hydrophilic DBAs. This water, derived from the pulpal chamber [12], may interfere with the subsequent coupling of the resin composite to DBAs, especially under simulated pulpal pressure [13,14]. Cadenaro et al. (2005) tested the effect of delayed polymerization on μTBS and adhesive layer and reported that the μTBS of HEMA-rich DBAs fell significantly when polymerization was delayed because more fluid could permeate the interface. Recent research revealed that water droplets, originating from a phase-separation reaction of HEMA-free DBAs, remain trapped in the adhesive layer after curing [11,15].

The aim of this study was to evaluate the effect of different application pulpal pressures on the μTBS of several DBAs to deep and superficial dentin samples when polymerization is not delayed. The null hypothesis was that positive pulpal pressure does not affect the bond strength of DBAs to dentin.

## 2. Materials and Methods

One hundred and fifty human extracted third molars were stored in 4 °C water for no more than one month. Crown segments were obtained by first removing the roots 1 mm beneath the cement-enamel junction (CEJ) using a slow-speed water-cooled diamond saw (Remet, Bologna, Italy). The occlusal enamel of each crown segment was subsequently removed with a parallel cut 1.5 mm above the CEJ for deep dentin or 2.5 mm for superficial dentin to expose the dentin surfaces. The exposed dentin was polished with 180 grit silicon carbide papers to create a standard bonding substrate in deep and peripheral dentin. Pulpal tissue was removed with a small forceps, taking care to avoid touching the pulp chamber walls. A pincer-type caliper was used for measurement of the remaining dentin thickness (RDT), which was between 0.5 to 0.9 mm for deep dentin and 1.5–1.8 mm for superficial dentin. Each sectioned tooth was attached to a Plexiglas platform (2 cm × 2 cm × 0.5 cm) that was perforated by an 18-gauge stainless steel tube using cyanoacrylate adhesive (ROCKET Heavy DVA, Corona, CA, USA). Each Plexiglas-tooth assembly was attached via polyethylene tubing to a 20-mL syringe barrel filled with distilled water in order to produce a hydrostatic pressure of 20 cm H_2_O at the dentin surface to be bonded.

### 2.1. Bonding Procedures

Five DBAs were examined in this study. They included one self-etching primer/adhesive system, Clearfil Protect Bond (Kuraray Medical Inc., Tokyo, Japan), three one-step self-etch adhesive systems, G-Bond (GC Corp., Tokyo, Japan), Clearfil S_3_-Bond (Kuraray Medical Inc., Tokyo, Japan), Bond Force (Tokuyama Corp., Tokyo, Japan), and a total-etch adhesive Scotchbond 1 XT (3M-ESPE, St Paul, MN, USA). Their compositions and pH values are listed in Table S1. Each DBA was applied according to the manufacturers’ instructions (Table S2). Light curing of the DBAs was performed using a halogen light-curing unit (XL-2500, 3M ESPE, St. Paul, MN, USA) with an output power intensity of 600 mW/cm^2^.

### 2.2. Microtensile Bond Strength Evaluation

Three different pulpal pressures were created:

1. Group A: Pulpal pressure was absent (0 cm H_2_O) during DBA application and composite build-up.
2. Group B: Pulpal pressure (20 cm H_2_O) was applied before DBA application and composite build-up.
3. Group C: Pulpal pressure was absent (0 cm H_2_O) during DBA application and curing. Pulpal pressure (20 cm H_2_O) was applied 3 s. after DBA curing and before composite build-up.

#### 2.2.1. Group A

In brief, in group A, the specimens were connected to a pulpal pressure device but no pulpal pressure was applied. Then, DBA and the flowable composite were polymerized.

#### 2.2.2. Group B

In group B, the specimens were exposed to pulpal pressure (20 cm H_2_O) 3 s after the DBA was then applied and cured. Then, the flowable composite was added and polymerized while the simulated pulpal pressure was maintained for 20 min.

#### 2.2.3. Group C

In group C, the specimens were connected to the pressure device, but the DBA was applied and cured without pulpal pressure (0 cm H_2_O). Three s after polymerization of the DBA, the bonded samples were exposed to 20 cm H_2_O pressure and composite build-up was realized. The simulated pulpal pressure was maintained for 20 min.

### 2.3. Build-Up, Samples Realization and Analysis

A 5-mm thick resin composite build-up was performed on the bonded dentin surfaces using a light-cured flowable resin composite (Gradia Direct LoFlo, GC Corp., Tokyo, Japan). Each composite-coupled specimen was sectioned perpendicular to the adhesive interfaces with a diamond saw under water cooling to produce resin-dentin slabs. Each slab was subsequently trimmed to produce resin-dentin beams with a cross-sectional area (measured with a digital caliper) of 1.0 mm^2^ at the bonded interface. Fifty teeth were used for each group and ten to twelve beams were obtained from each tooth. Samples were then divided into two subgroups according to residual dentin thickness (deep and superficial). The beams were then attached with cyanoacrylate adhesive to a testing jig, and loaded in tension with a universal testing machine (Bisco Inc., Schaumburg, IL, USA) at a crosshead speed of 0.9 mm/min until failure. The exact dimensions of each tested beam were measured with a digital micrometer.

After μTBS testing, the fractured sticks were analyzed by SEM (JSM-5200; JEOL, Tokyo, Japan) in order to evaluate the type of fracture between dentin and composite build-up. These samples were immediately fixed in 2.5% glutaraldehyde in 0.1 M cacodylate buffer pH 7.2 for 48 h and then rinsed several times with 0.1 M sodium cacodylate buffer. These were dehydrated in increasing concentrations of ethanol (50%, 70%, 80%, 90%, 95%, 100%) for 30 min each. The digitalized SEM images were subjected to quantitative image analysis using a digital slow-scan image recording system (SemAfore, JEOL, Sollentuna, Sweden).

The μTBS data were statistically analyzed by using a two-way ANOVA to test the effect of the DBAs and the simulated pulpal pressure conditions on bond strength. Distribution has been evaluated by a KS test.

## 3. Results

The μTBS results are summarized in Table 1 and Table 2.

In the absence of a pulpal pressure, Scotchbond 1 XT showed the highest μTBS of all tested adhesives when applied in deep dentin. Clearfil Protect Bond showed the highest μTBS of all the self-etching adhesives when applied in superficial dentin without pulpal pressure. Under the same conditions, Bond Force showed the lowest bond strength to both superficial and deep dentin.

Pulpal pressure application was responsible for a considerable reduction in μTBS in some but not all DBAs. When pulpal pressure was applied 3 s before the application of pulpal pressure (20 cm H_2_O), Clearfil Protect Bond and G-Bond both showed higher results than the other tested DBAs for both superficial and deep dentin and were not significantly different from each other. Under the same conditions, Bond Force showed the lowest μTBS. Microtensile bond strength results in deep dentin were significantly lower than superficial dentine for all tested DBAs. 

When pulpal pressure was applied 3 s after DBA curing (20 cm H_2_O), Scotchbond 1 XT showed significantly higher (*p* < 0.05) μTBS in both deep and superficial dentin. Under the same condition, Clearfil S_3_ and Bond Force showed significantly lower μTBS than other tested DBAs. Pulpal pressure was able to considerably reduce microtensile bond strength for both deep and for superficial dentin (Table 1 and Table 2), but it had a greater influence in deep dentin. 

**Figure 1:**
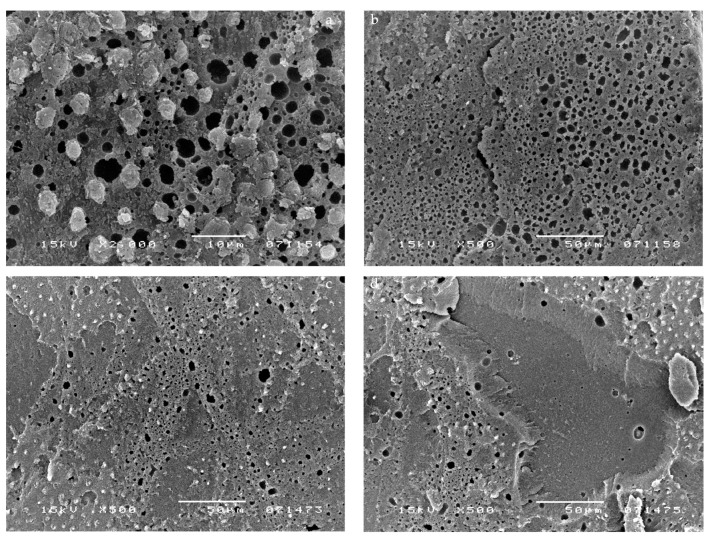
SEM photomicrographs illustrating the fractured surfaces for samples bonded with G-Bond. SEM analysis showed a large number of droplets within the adhesive layer of this DBA. Representative SEM images of G-Bond, applied without pulpal pressure (0 cm H_2_O), are shown in the (**a**,**b**) micrographs, where the presence of droplets at different levels throughout the adhesive layer can be seen (from 0.3 to 15 μm). (**c**,**d**) Micrographs show a representative sample of G-Bond applied simultaneously to pulpal pressure (20 cm H_2_O). Many droplets comparable with those in the (**a**,**b**) micrographs were observed (from 2 to 36 μm). Many droplets were displayed parallel to the scratches of the smear layer.

SEM micrographs showed a large number of voids within the adhesive layer of G-Bond (Figure 1). Representative SEM images of G-Bond, applied without pulpal pressure (0 cm H_2_O), are shown in Figure 1a,b. The presence of voids (ranging from 0.3 to 15 μm in diameter) at different levels throughout the adhesive layer can be seen. Figure 1c,d show representative samples of G-Bond that was applied when pulpal pressure was present. Many droplets, comparable with those in a and b micrographs, were observed (from 2 to 36 μm in diameter). Note that Figure 1b is taken at a lower magnification than Figure 1a. Figure 1c,d shows a representative sample where pulpal pressure was connected after G-Bond application and curing. Many droplets are displayed parallel to the scratches of the smear layer.

**Figure 2:**
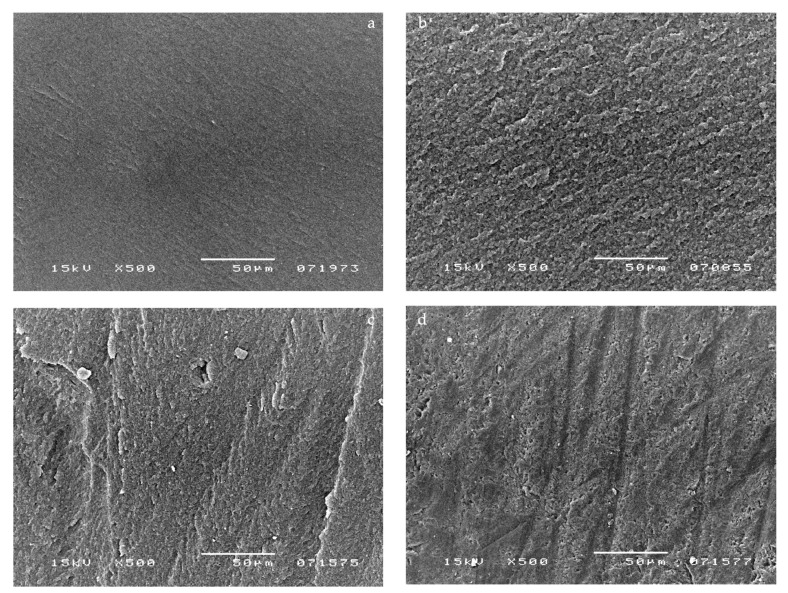
SEM photomicrographs illustrating fractured surfaces for samples bonded with Clearfil S_3_ Bond. SEM analysis showed a reduced number of small droplets within the adhesive layer of this DBA applied without pulpal pressure (0 cm H_2_O) (**a**). (**b**) Micrograph shows a representative sample of Clearfil S_3_ Bond applied simultaneously to pulpal pressure (20 cm H_2_O). In contrast to the 0 cm pulpal pressure group, almost the entire adhesive layer was affected by droplets. (**c**,**d**) Micrographs show the morphology of the adhesive applied and cured after the application of pulpal pressure (20 cm H_2_O). A reduced number of droplets was displayed.

SEM micrographs of failed bonds made with Clearfil S_3_ Bond showed few small droplets within the adhesive layer of this DBA applied without pulpal pressure (0 cm H_2_O) (Figure 2a). Figure 2b shows a representative sample of Clearfil S_3_ Bond when pulpal pressure was present. In contrast to the no pulpal pressure group, almost the entire adhesive layer was affected by voids. The micrographs in Figure 2c,d show the morphology of the adhesive layer when pulpal pressure was connected after DBA application and curing. A reduced number of voids was displayed.

**Figure 3:**
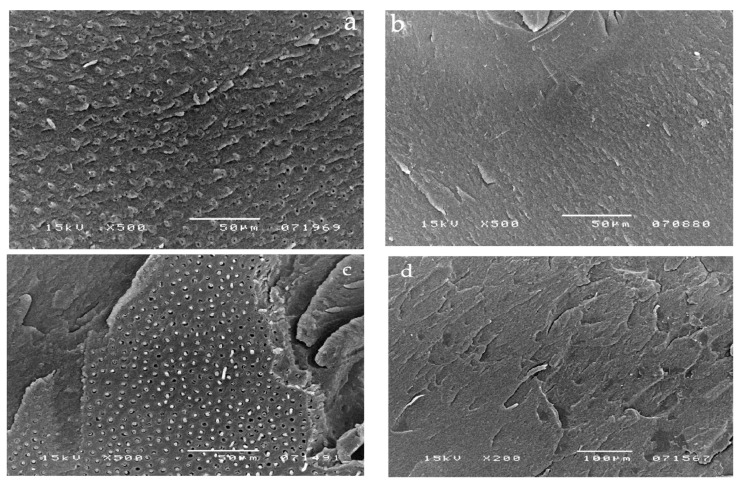
SEM photomicrographs illustrating the fractured surfaces for samples bonded with Clearfil Protect Bond. SEM analysis showed the absence of droplets within the adhesive layer of this DBA applied without pulpal pressure (0 cm H_2_O) (**a**) and simultaneously to pulpal pressure (20 cm H_2_O) (**b**). (**c**,**d**) micrograph show a representative sample of Clearfil Protect Bond applied and cured before the application of pulpal pressure (20 cm H_2_O). A high density of resin tags (cohesive fracture) and no droplets were displayed.

The Clearfil Protect Bond SEM micrographs show the absence of droplets within the adhesive layer of this DBA applied without pulpal pressure (0 cm H_2_O) (Figure 3a,b). The micrographs in Figure 3c,d showed a representative sample where pulpal pressure was inserted after Clearfil Protect Bond application and curing. A high density of resin tags and no droplets were displayed.

**Figure 4:**
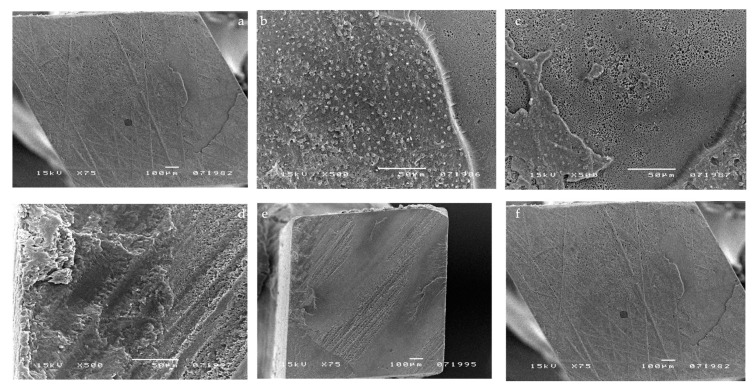
SEM micrographs illustrating fractured surfaces for samples bonded with Scotchbond 1 XT. Some resin tags were seen in the (**a**,**b**) micrographs when DBA was applied without pulpal pressure (0 cm H_2_O). (**c**,**d**) Micrographs show a representative sample of Scotchbond 1 applied simultaneously to pulpal pressure (20 cm H_2_O). A mixture of both small and large droplets (from 0.5 to 20 μm) was observed, which had sometimes coalesced to larger droplets. (**e**,**f**) Micrographs show a representative sample of Scotchbond 1 XT applied and cured before the application of pulpal pressure (20 cm H_2_O). A cohesive fracture and no droplets were present.

Scotchbond 1 XT micrographs showed some resin tags when DBA was applied without pulpal pressure (0 cm H_2_O) (Figure 4a,b). The micrographs in Figure 4c,d show a representative sample of Scotchbond 1 XT applied simultaneously to pulpal pressure (20 cm H_2_O). A combination of both small and large droplets (from 5 to 25 μm in diameter) was observed. They sometimes coalesced to larger droplets. Figure 4e,f shows a representative sample where pulpal pressure (20 cm H_2_O) was applied after Scotchbond 1 XT application and curing. A cohesive fracture and no droplets were present.

**Figure 5:**
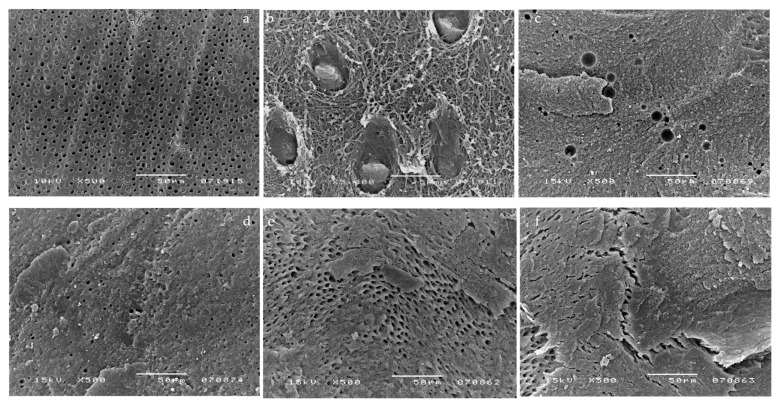
SEM photomicrographs illustrating fractured surfaces for samples bonded with Bond Force. (**a**,**b**) Micrographs show the morphology of Bond Force when applied without pulpal pressure (0 cm H_2_O) (mixed fracture). Several droplets and the delamination of the bonding layer were observed. (**c**,**d**) Micrographs show a representative sample of Bond Force applied during the application of pulpal pressure (mixed fracture). Almost the entire adhesive layer was affected by droplets and voids from 5 to 10 μm. (**e**,**f**) shows a representative sample of Bond Force applied and cured before the application of pulpal pressure (mixed fracture).

Bond Force micrographs show several droplets and fractures within the adhesive layer when it was applied without pulpal pressure (0 cm H_2_O) (Figure 5a,b). The micrographs in Figure 5c,d show a representative sample of Bond Force applied during the presence of pulpal pressure (20 cm H_2_O). Almost the entire adhesive layer was affected by droplets and voids (from 0.3 to 10 μm in diameter). Figure 5e,f shows a representative sample where pulpal pressure (20 cm H_2_O) was connected after DBA application and curing (mixed fracture).

## 4. Discussion

Pulpal pressure has been reported to influence dentine surface wetness and affect bond strength of previous generation DBAs [16]. The Periotron device was used to measure the surface wetness of dentin samples, supporting the concept that the dentin surface is wet, especially after smear layer removal and in the presence of a pulpal pressure [17]. Dentin wetness depends upon the remaining dentin thickness (RDT) and the presence or absence of a pulpal pressure [16,17].

The results of the current study showed that pulpal pressure significantly reduced the bond strengths of DBAs applied to dentine, and it had a greater influence in deep dentin probably because the deep dentin has a higher third conductance than superficial dentin [1,18,19,20].

To offer an explanation for these results, we speculate that after the application of DBAs on the dentin surface, solvent and/or water are incompletely evaporated, and become incorporated within the adhesive layer and remain trapped inside the polymerized film. When a simulated pulpal pressure is applied to dentin, an outward fluid flow from the dentinal tubules may occur across the smear layer, resulting in water flux through the dentinal tubules [21,22,23]. In specimens bonded without simulated pulpal pressure, the water in the adhesive film can be evaporated by an air blast before polymerization of the resin. However, specimens bonded under a simulated pulpal pressure dentinal fluid may replace the evaporated water and create a large number of voids (droplets) attracted by the presence of hydrophilic resin such as HEMA. 

The application of simulated pulpal pressure may increase fluid outward and create through and through water-channels within both the HL and the overlying adhesive film and at the interface between the DBA film and flowable composite [24,25]. These are potential sites of hydrolytic degradation that challenge the longevity of restorations [26]. HEMA-based DBAs are prone to hydrolytic degradation, resulting in the reduction in their mechanical properties [26,27]. However, G-Bond was unaffected by the presence of pulpal pressure in all tested conditions, as confirmed by the literature [13]. It is the only HEMA-free tested DBA that contains 4-MET, a less reactive hydrophilic monomer compared with HEMA. Interesting SEM micrographs displayed the presence of voids at different levels throughout the G-Bond layer, even without the application of pulpal pressure (Figure 1a,b), indicating that they come from water within G-Bond. Phase separation of water from the adhesive was probably responsible for the appearance of the voids. In contrast, HEMA-based tested DBAs did not show phase separation, as demonstrated by a recent study [15]. That is, in the presence of HEMA, unevaporated water can dissolve into HEMA, with which it is miscible. SEM micrographs of HEMA-based DBAs without the application of pulpal pressure confirmed the absence or the reduced number of droplets. The μTBS of HEMA-based DBAs dropped significantly when pulpal pressure was applied, especially for one-step self-etch adhesives (Table 1 and Table 2). The μTBS results can be clearly correlated to the increased number of droplets displayed in the SEM micrographs (Figure 2b–d, Figure 3c,d, Figure 4c,d and Figure 5c,d).

Bond strength of G-Bond was lower than HEMA-based tested DBAs. When pulpal pressure was applied, the number of voids in G-Bond did not change and there was no drop in bond strength. The lack of more voids in G-Bond when pulpal pressure was applied indicates that G-Bond droplets have a different origin and should be attributed to a phase-separation when the more volatile acetone evaporates faster than water. The residual water is not miscible with the remaining monomers. Of interest is the fact that the presence of these water-filled voids did not cause a noticeable drop of μTBS (Table 1 and Table 2; Figure 1b,d).

Clearfil Protect Bond showed the highest μTBS of the self-etching adhesives both with or without pulpal pressure, probably because its solvent-free hydrophobic layer may prevent water uptake and droplet formation. Moreover, since it is a weakly acidic self-etch primer, the smear layer is not completely removed but is modified, causing a lower increase in dentin permeability and reducing the risk of water contamination of the dentin surface [15].

Scotchbond 1 XT showed both the highest μTBS of all the tested adhesives and the highest decrease in μTBS when pulpal pressure was applied, probably because dentin etching was responsible for complete smear layer removal, increasing the permeability of dentin, and permitting a higher water flux through dentinal tubules [27]. Etch and rinse adhesives such as Scotchbond1 XT are sensitive to hydrolytic degradation because the exposure of the collagen matrix of dentin by acid etching may activate matrix metalloproteinase (MMPs), which are known to cause collagenolysis in the presence of water. Nanoleakage due to incomplete resin penetration in collagen network is considered as pathways for degradation of the adhesive interfacial region through water permeation into the hybrid layer [28]. The degradation has also been shown to result in a significant decrease in bond strength over time after water storage, as demonstrated in other studies [29].

Time of polymerization and amount of energy influence the water flux from dentinal tubules because of its relation with the degree of conversion [30]. In fact, the presence of unpolymerized hydrophilic resin in the DBA thickness may increase the water uptake, so it is possible that more rapid and more complete curing may create a less permeable resin film that reduces water uptake.

Water uptake may be responsible for the elution of unreacted monomers in the thickness of DBAs that promote plasticization of methacrylic polymers [31,32,33,34,35,36].

Apart from its chemical composition and the presence of less hydrophilic co-monomers, the G-Bond results may be attributed to its mode of application. In fact, it needs a strong air-blast to remove the solvent. This procedure might improve the resin–collagenic network infiltration. Since a strong or longer air-blast reduces the permeability of adhesive resin films [33], they also might reduce the permeability of resin–dentin bonds and prevent water contamination of the adhesive layer.

This study suggests that HEMA-based one-step self-etch systems are more sensitive to pulpal pressure and water uptake than HEMA-free one-step self-etch systems. In spite of the good behavior of G-Bond, SEM micrographs and μTBS results showed that two-step self-etch systems, such as Clearfil Protect Bond, represent a better choice in adhesive dentistry. The hydrophobic layer applied on primed dentin is able to prevent water uptake from dentinal tubules, resulting in being free from many voids/droplets that may greatly affect the longevity of bonding. 

G-Bond was only partially affected by pulpal pressure, but these encouraging results need to be confirmed with other investigations. Evaluating how pulp pressure affects bond strength in different types of DBAs applied in deep dentin is one of the main limitations of this study, and it could be considered for further analysis. Certainly, it could be very useful to test other adhesive systems with different conditions of the dentin and pressure of the latter.

The study demonstrated that in vitro simulated pulpal pressure adversely affected bonding of DBAs to coronal dentin. Therefore, the null hypothesis that positive pulpal pressure does not affect the bond strength of DBAs has to be rejected.

## Figures and Tables

**Table 1:** Microtensile bond strengths (means ± standard deviations) of the DBAs bonded to deep dentin.

Materials	No Pulpal Pressure (MPa)	Pulpal Pressure Applied 3 s before DBA Curing (MPa)	Pulpal Pressure Applied 3 s after DBA Curing (MPa)
	Deep Dentin		
G-BOND	19.1 ± 7.1	16.9 ± 5.2	16.7 ± 4.2
Clearfil S_3_	23.1 ± 8.4	11.0 ± 5.8	11.8 ± 6.1
Protect Bond	28.5 ± 12.0	19.3 ± 6.9	19.6 ± 5.5
Scotchbond 1 XT	29.1 ± 12.3	12.2 ± 4.6	21.4 ± 9.2
Bond Force	17.8 ± 6.9	6.6 ± 3.2	11.3 ± 5.8

Values are mean ± SD microtensile bond strength in MPa.

**Table 2:** Microtensile bond strengths (means ± standard deviations) of the DBAs bonded to superficial dentin.

Materials	No Pulpal Pressure (MPa)	Pulpal Pressure Applied 3 s before DBA Curing (MPa)	Pulpal Pressure Applied 3 s after DBA Curing (MPa)
	Superficial Dentin		
G-BOND	22.6 ± 5.5	21.7 ± 7.2	20.8 ± 6.6
Clearfil S_3_	22.4 ± 7.9	14.4 ± 7.3	16.1 ± 7.9
Protect Bond	30.1 ± 12.3	21.7 ± 5.5	22.1 ± 6.4
Scotchbond 1 XT	37.6 ± 7.5	16.1 ± 5.2	26.3 ± 12.3
Bond Force	18.6 ± 7.2	7.5 ± 2.6	12.8 ± 7.3

Values are mean ± SD microtensile bond strength in MPa.

## Data Availability

Data are available on request to M.M.

## Supplementary Materials

The following are available online at https://www.mdpi.com/article/10.3390/ma14206200/s1, Table S1: List of dentin bonding agents (DBAs) investigated, their compositions and pH values, Table S2: Application procedures for the four DBAs investigated in the study.
