# Supplementary material for: The Effect of Different Condition of Pulpal Pressure on Microtensile Bond Strength of Several Dentin Bonding Agents on Deep and Superficial Dentin
Source: Materials (Basel). 2021 Oct 19;14(20):6200. doi: 10.3390/ma14206200 (PMC8541316; doi:10.3390/ma14206200)
Supplement: Supplementary file 1 [file materials-14-06200-s001.zip › materials-1412709-supplementary.pdf]

Table S1. List of dentin bonding agents (DBAs) investigated, their compositions and pH values.

DBAs	Chemical Composition	pH Value
Clearfil S_3-Bond<br><br>Kuraray Medical Inc., Tokyo, Japan	2-hydroxyethyl methacrylate<br>Bis-phenol A diglycidylmethacrylate<br>10-Methacryloyloxydecyl dihydrogen phosphate<br>Silanated colloidal silica<br>dl-Camphorquinone<br>Ethyl alcohol<br>Water	2.7
G-Bond<br><br>GC Corp., Tokyo, Japan	4-methacryloxyethyltrimellitate anhydride<br>Triethylene glycol dimethacrylate<br>Urethane dimethacrylate<br>Acetone<br>Water	>2
Clearfil Protect Bond<br><br>Kuraray Medical Inc.	10-Methacryloyloxydecyl dihydrogen phosphate<br>Bis-phenol A diglycidylmethacrylate<br>2-hydroxyethyl methacrylate<br>Hydrophobic dimethacrylate<br>dl-Camphorquinone<br>N,N-Diethanol-p-toluidine<br>Silanated colloidal silica<br>Surface treated sodium fluoride	~2
Scotchbond 1 XT<br>3M-ESPE, St Paul, USA	Bis-GMA<br>2-hydroxyethyl methacrylate<br>Hydrophobic dimethacrylate<br>Polyalkenoic acid copolymer<br>Ethyl alcohol<br>Water	5
Bond Force<br>Tokujama Dental, Tokyo, Japan	AlcoholC2-4 alkil<br>Methacryloyloxyalkyl acid phosphate<br>2-Hydroxyethyl methacrylate<br>Bis-GMA<br>Triethylene glycol dimethacrylate<br>Camphorquinone<br>Purified water	2.3

Table S2. Application procedures for the four DBAs investigated in the study.

DBAs	Application Mode	Adhesive Classification
G-Bond	The DBA was applied in one coat using the micro-tip applicator and left undisturbed for 10 sec. It was dried thoroughly for 5 sec with oil-free air under maximum air pressure. Then it was light-cured for 20 sec.	One-step<br>self-etching system
Clearfil S_3-Bond	The DBA was applied to dentin for 20 sec. It was air dried for 5 sec to evaporate the solvent. Then it was light-cured for 20 sec.	One-step<br>self-etching system
Clearfil Protect Bond	Primer was applied in two consecutive coats and left undisturbed for 20 sec, then the solvent was evaporated for 10 sec.	Two-step<br>self-etching system

	Bonding was applied in two consecutive coats and spread using a gentle stream of oil free air for 2 sec. Then it was light cure for 20 sec.	
Scotchbond 1 XT	DBA was applied in two consecutive coats and spread using a gentle stream of oil free air for 5 sec. to evaporate the solvent. Then it was light-cured for 20 sec	Two-step total etch system
Bond Force	The DBA was applied to dentin for 20 sec. It was air dried for 5 sec to evaporate the solvent. Then it was light-cured for 20 sec.	One-step self-etching system
